# *ELOVL6*: A Key Factor in the Gender Differences of Olanzapine-Induced Weight Gain

**DOI:** 10.3390/ph19081223

**Published:** 2026-08-04

**Authors:** Keke Che, Dejuan Li, Lu Liu, Yanyan Zhang, Xuemei Liu, Changhua Hu

**Affiliations:** 1 College of Pharmaceutical Sciences, Southwest University, Beibei District, Chongqing 400715, China; beayerchoe@163.com; 2 Department of Pharmacy, Chongqing Academy of Medical Sciences, Chongqing General Hospital, Chongqing University, No. 118, Xingguang Avenue, Liangjiang New Area, Chongqing 401147, China; 3 School of Pharmaceutical Sciences, Chengdu Medical College, No. 783, Xindu Avenue, Xindu District, Chengdu 610500, China; 102022031@cmc.edu.cn; 4 School of Mental Health, North Sichuan Medical College, Maoyuan South Road, Shunqing District, Nanchong 637100, China; liuludoctor88@nsmc.edu.cn; 5 School of Biomedical Engineering, Chongqing Medical University, No. 1, Yixueyuan Road, Yuzhong District, Chongqing 400016, China; yzhangtmmu@163.com

**Keywords:** olanzapine, antipsychotic, weight gain, *ELOVL6*, long-chain fatty acid

## Abstract

**Background:** This study investigates the role of *ELOVL6* in the weight gain induced by olanzapine and the mechanisms underlying the female-specific response. **Methods:** An olanzapine-induced weight gain model in SD rats was developed. Transcriptome sequencing and metabolomics identified genes and metabolites linked to female-specific weight gain. Validation was conducted in HL7702 cells, and DNA pulldown, ChIP-qPCR, and molecular docking explored the molecular mechanism. A genetic polymorphism association study was also performed. **Results:** Female rats showed more pronounced weight gain compared to males following olanzapine treatment. Transcriptomic and metabolomics analyses revealed that olanzapine significantly upregulates hepatic *ELOVL6* expression and long-chain fatty acid synthesis in females. Inhibition of *ELOVL6* restores the elevated levels of the lipid, *SREBP-1c* and *FAS* induced by olanzapine. The transcription factor *HNF-3α* is implicated in *ELOVL6* regulation, and some olanzapine metabolites can directly interact with *HNF-3α*. Furthermore, a single-nucleotide polymorphism (SNP) at the *ELOVL6* promoter -1427 bp correlates with weight gain in female patients. **Conclusions:** Olanzapine metabolites interact with *HNF-3α*, enhancing *ELOVL6* transcription. The upregulated *ELOVL6* promotes long-chain fatty acid synthesis via the *SREBP-1c*/*FAS* pathway, contributing to female-specific weight gain.

## 1. Introduction

Schizophrenia (SCZ) is a chronic and disabling mental illness with core symptom domains involving positive symptoms (e.g., hallucinations, illusions, and thought disorder), negative symptoms (such as social isolation, flattened affect, and diminished motivation), and impairments in learning and thinking [1,2,3]. Its onset most often occurs from late adolescence through young adulthood. Affected individuals often face significant social challenges, such as stigmatization, marital difficulties, social isolation, and an elevated risk of suicide, thereby posing a major public health burden. According to a report by the Lancet Psychiatry Commission, approximately 23 million individuals worldwide are affected by SCZ, contributing to a substantial socioeconomic burden [4,5].

Olanzapine (Olan), a second-generation antipsychotic agent, functions as a non-selective antagonist at dopamine D_2_ and serotonin (5-HT) receptors [6]. It is widely prescribed for schizophrenia, acute manic episodes in bipolar disorder, and maintenance treatment, demonstrating a robust curative effect [7]. Characterized by a broad therapeutic window, favorable tolerability, and enhanced adherence profiles, Olan has received Food and Drug Administration approval for treating schizophrenia and bipolar disorder in adults and adolescents aged ≥13 years, ranking among the most frequently prescribed antipsychotics worldwide [8]. However, chronic administration, akin to other antipsychotics, precipitates substantial weight gain and metabolic derangements, fostering metabolic syndrome marked by obesity and insulin resistance, thereby significantly elevating risks for type 2 diabetes and cardiovascular disease [9]. A well-documented dose–response relationship exists between Olan and adiposity, with pronounced effects observed in first-episode psychosis and early-stage schizophrenia [10,11,12,13].

Importantly, Olan-induced weight gain exhibits marked sexual dimorphism. Previous studies have demonstrated that females experience significantly greater incidence and severity of weight gain compared with males, as reflected by steeper body mass index (BMI) trajectories and accentuated central adiposity [14,15]. Given the chronic or lifelong treatment requirements in schizophrenia, Olan-induced weight gain critically undermines therapeutic adherence; notably, females demonstrate elevated rates of self-discontinuation driven by weight-related distress, culminating in relapse, symptom exacerbation, and increased rehospitalization, representing a major limitation in the clinical practice of Olan [16].

Currently, the common clinical approaches to mitigate the weight-gain side effects of Olan predominantly involve dietary restrictions and exercise [17]. These methods increase the patient’s effort and reduce treatment adherence, while failing to directly address this side effect. In more severe cases, alternative strategies involve reducing the medication dosage or combining Olan with other drugs such as metformin or topiramate to counteract its metabolic side effects [18]. However, these approaches often represent off-label use, potentially affecting the treatment of SCZ and increasing the risk of other side effects. Consequently, a viable solution for Olan-induced weight gain is lacking, highlighting the necessity to understand the molecular mechanisms underlying this side effect. Current evidence suggests that Olan-induced weight gain involves multiple central and peripheral pathophysiological pathways. In the central nervous system, it is primarily associated with hypothalamic feeding center disruptions. Olan can antagonize the serotonin 2C (5-HT2C) receptor, impairing its inhibition of the ghrelin receptor *GHSR1a*, thereby enhancing downstream *GHSR1a* signaling and increasing hypothalamic orexigenic neuropeptide (NPY) levels, which promote increased food intake behavior [19]. Additionally, Olan can also trigger increased feeding through the *Nr5a2*/*Agrp* pathway in the arcuate nucleus of the hypothalamus [20]. As for the peripheral system, Olan is known to directly induce metabolic dysregulation by promoting adipocyte differentiation, enhancing glycolysis, and increasing glucose uptake, thereby stimulating lipid storage and adipogenesis [21]. Furthermore, Olan can cause gut microbiota dysbiosis, leading to obesity through altered metabolism [22]. These findings suggest that Olan primarily promotes weight gain by influencing central appetite pathways and peripheral metabolic regulation. However, existing mechanistic investigations predominantly employ single-sex or mixed-sex cohorts, inadequately addressing sexual dimorphism and leaving female-specific pathophysiological mechanisms largely unexplored.

Accordingly, this study leverages sexual dimorphism in Olan-induced weight gain as a mechanistic entry point, utilizing chronic administration models in both sexes to systematically characterize sex-dependent alterations in energy metabolism, adipose tissue physiology, and central appetite regulation. The primary objectives are to elucidate the sex-specific molecular mechanisms underlying Olan-induced adiposity, identify critical molecular targets that confer heightened vulnerability to metabolic adverse effects in female patients, and establish a mechanistic framework for the development of targeted therapeutic interventions and the implementation of individualized precision medicine in schizophrenia management. This investigation holds substantial translational value for enhancing treatment adherence and improving long-term prognostic outcomes, with significant implications for both clinical practice and public health.

## 2. Results

### 2.1. Upregulated ELOVL6 Gene Expression in Liver Tissues Is Involved in Fat Synthesis

**Figure 1:**
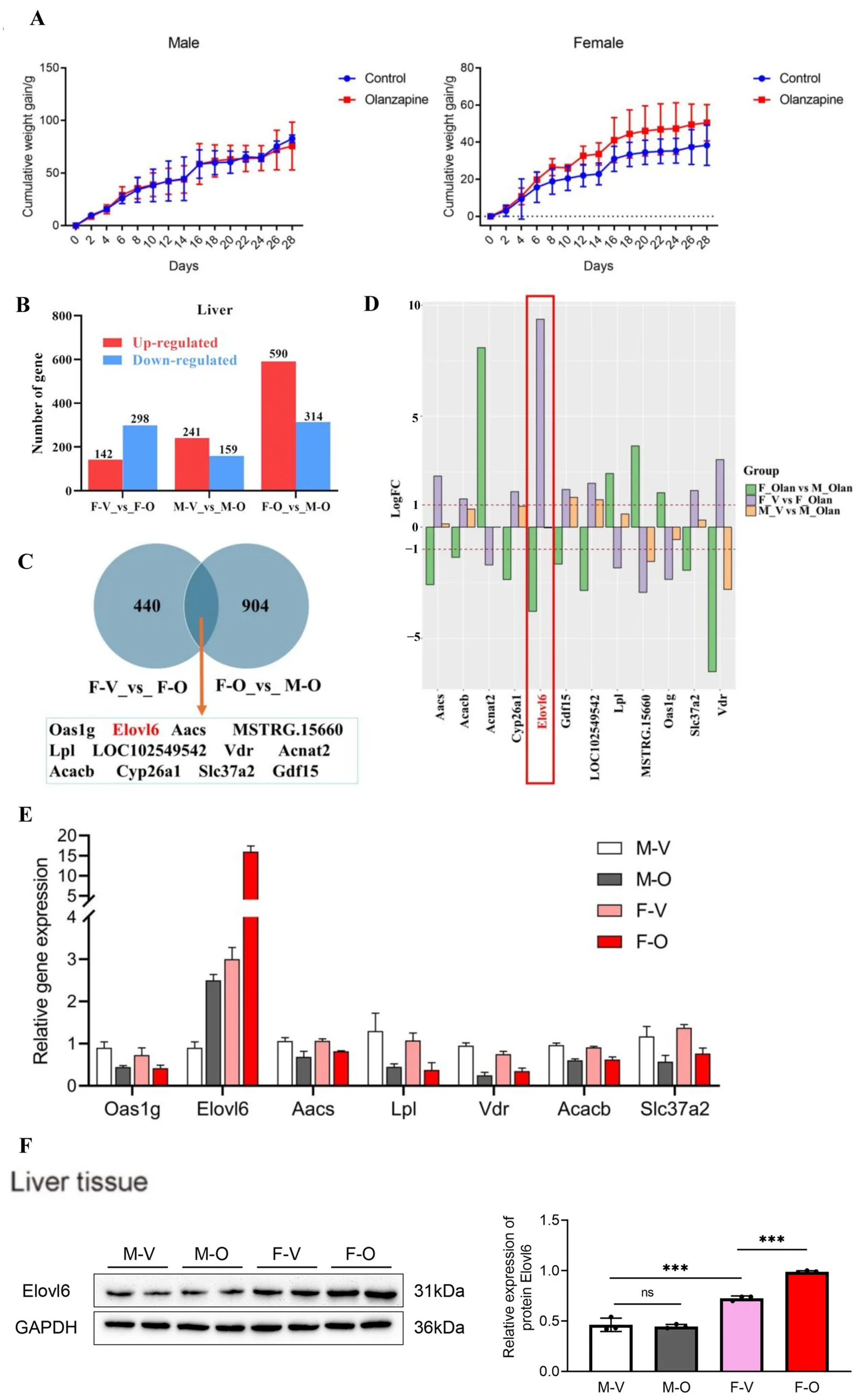
Sex-specific effects of olanzapine treatment on rat body weight gain and analysis of differentially expressed genes (DEGs) in liver tissue. (**A**) Effect of olanzapine treatment on body weight gain in male and female rats (*n* = 6). (**B**) Statistical results of DEGs in pairwise comparisons. (**C**) Venn diagram of DEGs between the F_V vs. F_O and F_O vs. M_O comparisons. (**D**) Expression levels of selected DEGs. (**E**) qPCR validation of selected gene expression (*n* = 3). (**F**) Western blot and qPCR validation of *Elovl6* expression (*n* = 3). *** *p* < 0.001, ns represents not significant. F: female; M: male; V: vehicle (control); O: olanzapine; FC: fold change.

The effects of Olan on body weight in male and female rats were evaluated in a 28-day administration study. Both male and female rats treated with Olan exhibited varying degrees of weight gain relative to controls. Specifically, no obvious changes in body weight were observed in male rats, whereas female rats demonstrated significant weight gain (Figure 1A) (*p* < 0.05). These results indicate pronounced sexual dimorphism in Olan-induced weight gain, with female rats being more susceptible.

To investigate the molecular mechanisms underlying this sexual dimorphism, transcriptome sequencing was performed. Gene expression levels were quantified, followed by differential expression and enrichment analyses. FPKM density distribution and inter-sample correlation analyses confirmed data reliability and sample suitability. Figure 1B presents the statistical analysis of DEGs in liver tissue. The intersecting DEGs screened from the “F_V vs. F_O” and “F_O vs. M_O” (F: female, M: male, V: vehicle, O: Olan) comparisons mainly included *Oas1g*, *Elovl6*, *Aacs*, and *MSTRG.15660*, *Lpl*, *LOC102549542*, *Vdr*, *Acnat2*, *Acacb*, *Cyp26a1*, *Slc37a2*, and *Gdf15* (Figure 1C), with their expression patterns displayed in Figure 1D,E. Western blot validation revealed that *ELOVL6* protein expression in rat liver tissue was consistent with the qPCR results (Figure 1F). These results indicate that *ELOVL6* expression was significantly elevated in Olan-treated female rats compared to both control and Olan-treated male rats. This finding identified *ELOVL6* as a key candidate gene mediating sex-specific differences in Olan-induced hepatic lipid metabolism.

**Figure 2:**
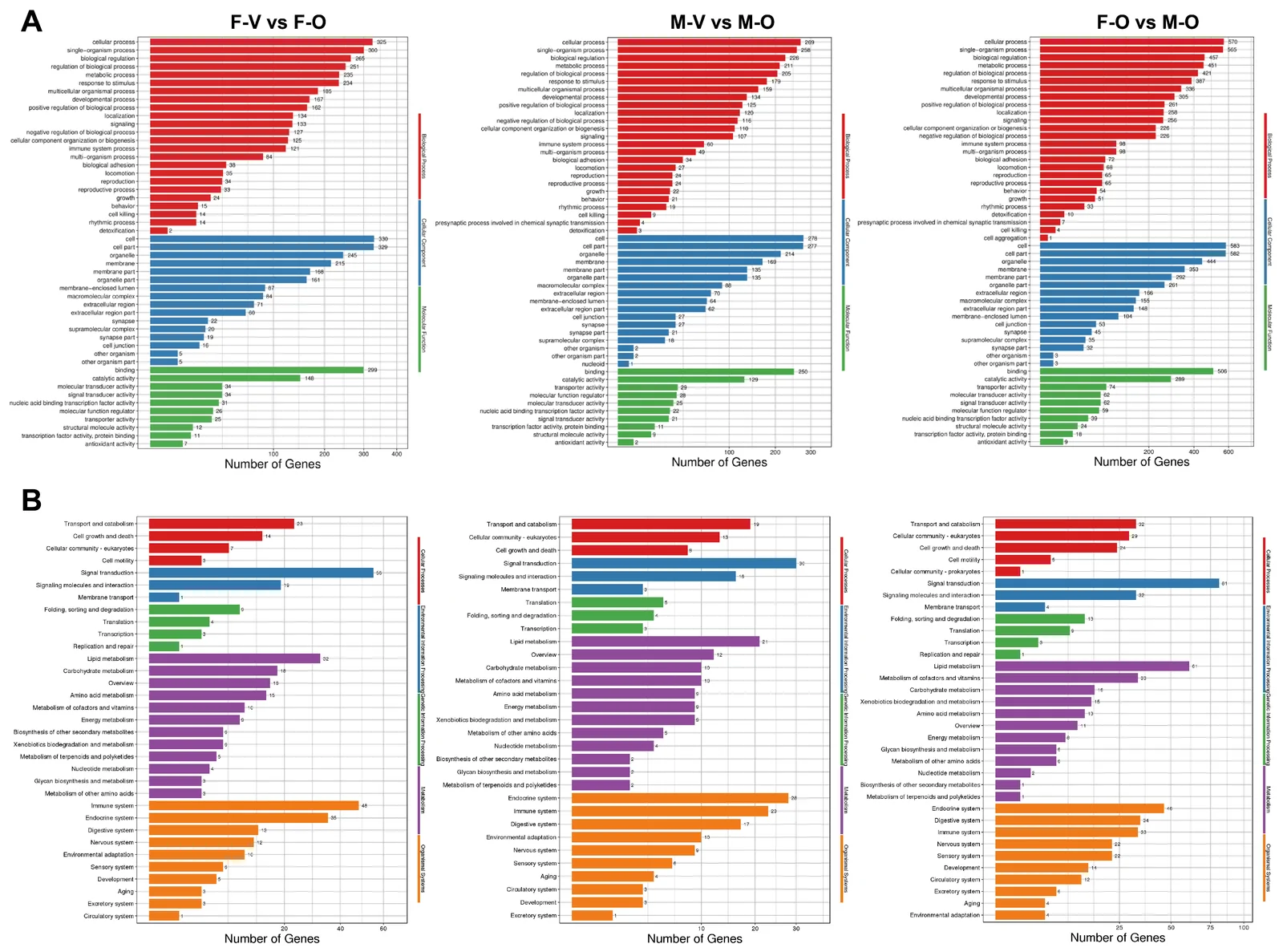
GO and KEGG enrichment analysis of DEGs. (**A**) GO statistical histogram of DEGs in liver tissue. (**B**) KEGG statistical histogram of DEGs in liver tissue. From left to right, panels represent the F_V group vs. F_O group, M_V group vs. M_O group, and F_O group vs. M_O group, respectively.

Subsequently, the biological functions of DEGs were elucidated through GO and KEGG analyses. Figure 2A shows the most significantly enriched terms. Additionally, the top 30 KEGG pathways are shown (Figure 2B). These analyses revealed that differentially expressed pathways were predominantly associated with lipid metabolism, carbohydrate metabolism, and energy metabolism. Notably, in the comparison between vehicle-treated and Olan-treated females (F-V vs. F-O), *ELOVL6* was enriched in lipid metabolism-related pathways, including fatty acid elongation, fatty acid metabolism, and biosynthesis of unsaturated fatty acids. These results suggest that Olan may promote hepatic lipid synthesis through *ELOVL6*-mediated fatty acid elongation.

**Figure 3:**
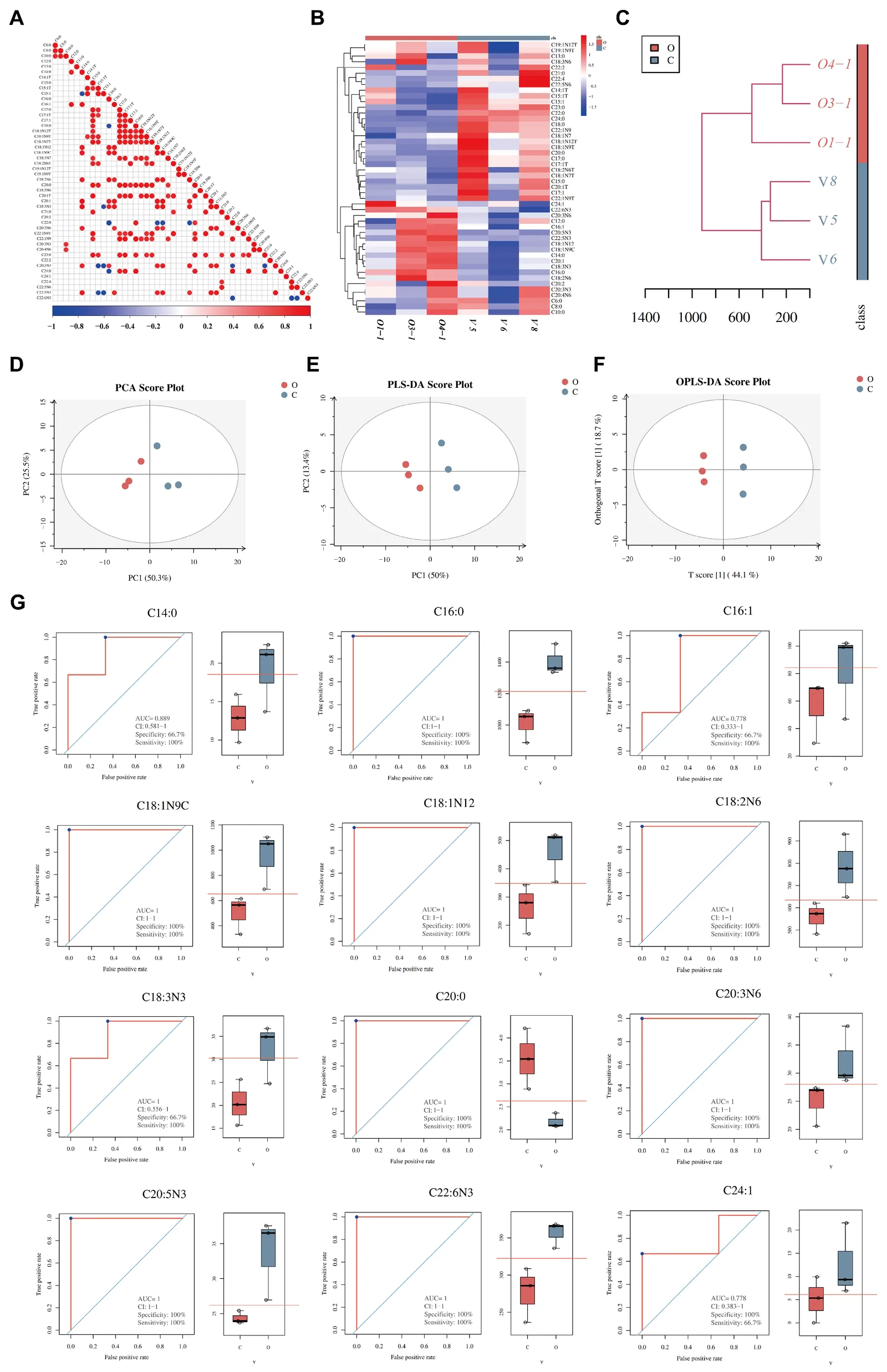
Fatty acid metabolomics of liver tissue. (**A**) The correlation heat map of differential metabolites: both ordinates and oblique ordinates represent the names of the differential metabolites. The color represents correlation; red represents positive correlation, blue represents negative correlation, and the darker the color, the greater the correlation. (**B**) Cluster heat map of differential metabolites, in which the relative content was shown by color difference. Columns represent samples, rows represent metabolite names, and the cluster tree on the left of the figure is the differential metabolite cluster tree. (**C**) Overall tree of differential metabolites. (**D**) Principal component analysis (PCA). (**E**) Partial least squares-discriminant analysis (PLS-DA). (**F**) Orthogonal partial least squares-discriminant analysis (OPLS-DA). (**G**) Differentially high expression of fatty acids in the Olan group and their corresponding receiver operating characteristic curve (ROC) (*n* = 3). AUC: area under the curve; CI: confidence interval; C: control, O: olanzapine.

To validate these transcriptomic findings, targeted fatty acid metabolomics analysis was conducted. Hierarchical clustering of metabolite relative quantification values demonstrated marked differences between the Olan-treated and control rats (Figure 3B). Distance matrices were calculated, and hierarchical clustering generated a dendrogram illustrating inter-sample similarity (Figure 3C). Multivariate statistical analysis demonstrated significant separation in hepatic fatty acid metabolite profiles between groups, indicating substantial differences in fatty acid composition and concentration (Figure 3D–F). Collectively, these results demonstrate that Olan significantly alters the hepatic fatty acid metabolic profile, particularly promoting long-chain fatty acid accumulation.

Quantitative analysis of fatty acid metabolites confirmed these findings (Figure 3G). Normalized intensities of multiple fatty acid metabolites—including C16:0, C16:1, C18:1n9c, C18:1n12, C18:2n6, C18:3n3, C20:1, C20:3n6, C20:5n3, C22:6n3, and C24:1—were markedly elevated following Olan treatment. The receiver operating characteristic (ROC) curve demonstrated area under the curve (AUC) values approaching 1.0, with 100% predictive sensitivity, suggesting that these metabolites may serve as highly accurate biomarkers for Olan-induced metabolic alterations. These findings support the role of *ELOVL6*-encoded enzyme in synthesizing long-chain fatty acids (>16 carbons), which are critical for Olan-mediated lipid synthesis. Therefore, integrating transcriptomic and metabolomic evidence, this study establishes *ELOVL6* as the central target for subsequent mechanistic investigations.

### 2.2. Olan Treatment Promotes Hepatic Steatosis via ELOVL6-Mediated Lipogenesis

Previous animal experiments demonstrated that Olan induced excessive weight gain in female rats, accompanied by hepatic upregulation of *ELOVL6* and long-chain fatty acid accumulation. Histological examination revealed dose-dependent hepatic steatosis in both sexes, characterized by lobular disarray, central vein congestion, and inflammatory infiltration, with females exhibiting more severe damage, including focal hepatocellular necrosis (see Supplementary Materials). KEGG enrichment analysis revealed that differential *ELOVL6* expression was significantly linked to the pathways of fatty acid elongation, fatty acid metabolism, and unsaturated fatty acid biosynthesis. Given that *ELOVL6* encodes a long-chain fatty acid elongase, these results suggest a potential role for *ELOVL6* in mediating Olan-induced lipogenesis effects. Accordingly, the present study established stable *ELOVL6* overexpression and knockdown cell lines in vitro to elucidate *ELOVL6*’s functional role in Olan-regulated lipogenesis.

**Figure 4:**
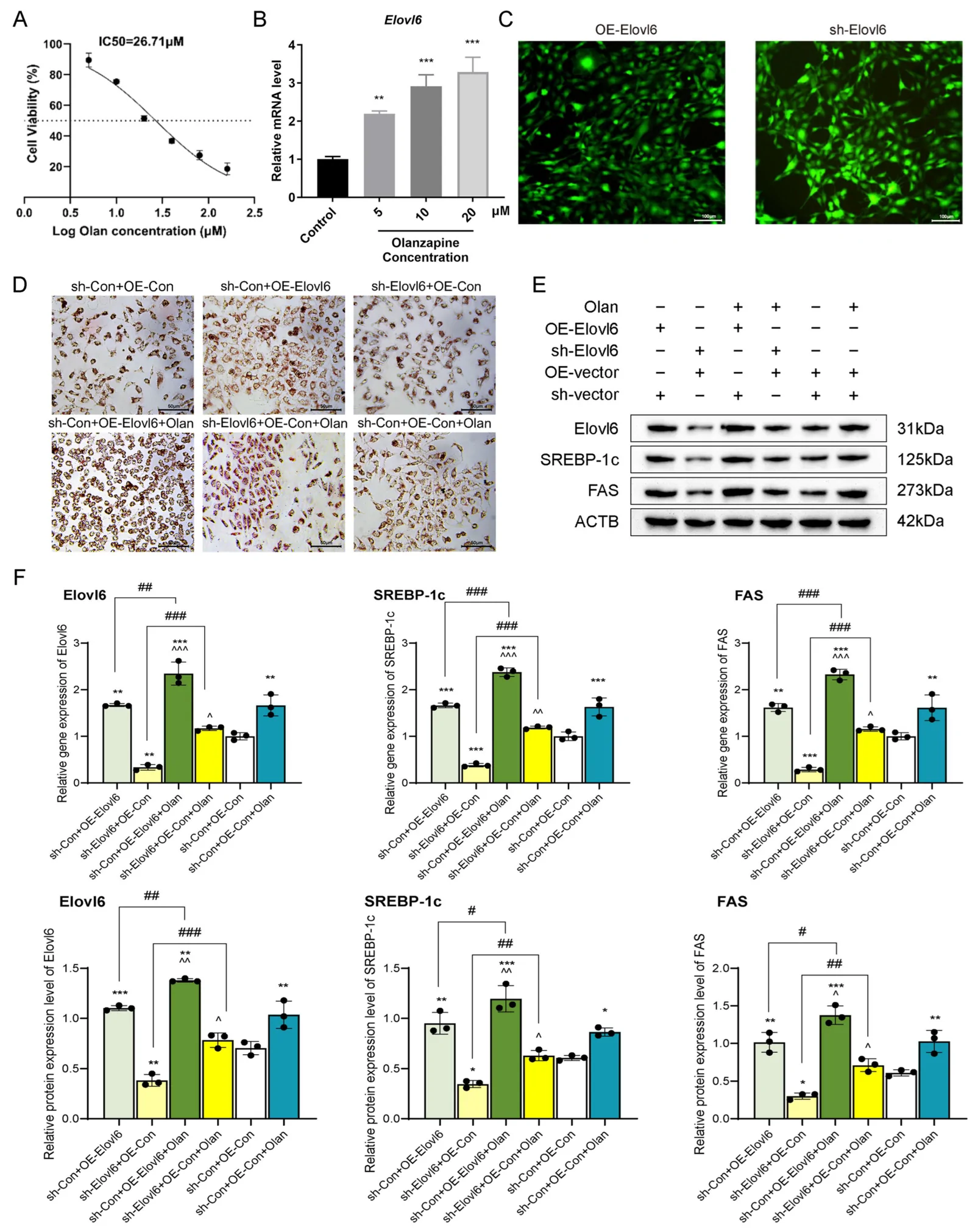
The *ELOVL6* gene regulates hepatic lipid synthesis. (**A**) Toxicity curve of Olan on HL7702 cells (*n* = 3). (**B**) Effect of different Olan concentrations on *ELOVL6* gene expression (*n* = 3); Compared with the Control group, ** *p* < 0.01, *** *p* < 0.001. (**C**) Expression of GFP in cells infected with lentivirus. (**D**) Oil Red O staining to detect lipid synthesis in hepatocytes from different groups (400×). (**E**) Detection of the *ELOVL6* gene and fatty acid synthesis-related gene (*SREBP-1c*, *FAS*) levels in different groups. (**F**) Detection of *ELOVL6*, *SREBP-1c*, and *FAS* protein levels in different groups (*n* = 3). *, ^ denote statistically significant differences compared to the sh-Con + OE-Con group, and the sh-Con + OE-Con + Olan group, respectively. *, #, ^ represent *p* < 0.05; **, ##, ^^ represent *p* < 0.01; ***, ###, ^^^ represent *p* < 0.001. oe: overexpression. sh: short hairpin.

To determine the optimal Olan concentration, cytotoxicity and gene induction effects were first assessed. As shown in Figure 4A, Olan inhibited HL7702 cell viability in a dose-dependent manner, with a 24 h IC_50_ of 26.71 μM. According to these results, 1 μM (low dose), 10 μM (>75% cell viability), and 20 μM (approaching IC_50_) were selected for qPCR validation. Olan upregulated *ELOVL6* mRNA expression dose-dependently, with 20 μM inducing the strongest effect (approximately 3-fold vs. control; Figure 4B). Thus, 20 μM was selected as the working concentration, balancing gene induction potency against cellular viability. Stable *ELOVL6* knockdown and overexpression HL7702 cell lines were generated using pLVX-shRNA1 and pCDH-CMV-MCS-EF1-copGFP-T2A-Puro lentiviral vectors, respectively, and GFP fluorescence confirmed successful transduction (Figure 4C).

Oil Red O staining validated both the regulatory role of *ELOVL6* in lipogenesis and the modulatory effects of Olan (Figure 4D). Relative to controls (sh-Con + OE-Con), *ELOVL6* overexpression (sh-Con + OE-*ELOVL6*) increased lipid content, whereas the stable interference group of *ELOVL6* (sh-*ELOVL6* + OE-Con) decreased it, confirming that *ELOVL6* promotes hepatocellular lipogenesis. Furthermore, Olan partially reversed the lipid-lowering effect of *ELOVL6* knockdown while further enhancing lipid accumulation in the overexpression group, indicating that Olan promotes lipogenesis through an *ELOVL6*-dependent pathway.

At the molecular level, qPCR and Western blot analyses confirmed these findings. As shown in Figure 4E, the stable interference group (sh-*ELOVL6* + OE-Con) significantly downregulated mRNA levels of *ELOVL6*, *SREBP-1c*, and *FAS*, whereas overexpression (sh-Con + OE-*ELOVL6*) significantly upregulated all three (*p* < 0.01). Notably, Olan treatment further upregulated the expression of *ELOVL6*, *FAS*, and *SREBP-1c* in both genetic backgrounds (*p* < 0.01). Protein expression patterns mirrored transcriptional trends (Figure 4F): stable *ELOVL6* knockdown significantly downregulated all three proteins, whereas overexpression significantly upregulated them (*p* < 0.05). Consistently, Olan enhanced protein levels in the overexpression group and partially reversed the downregulation in the knockdown group cells (*p* < 0.05). Collectively, these results demonstrate that Olan activates the *SREBP-1c*–*FAS* axis through an *ELOVL6*-dependent pathway, thereby promoting hepatocellular lipogenesis and establishing *ELOVL6* as a critical effector in Olan-mediated hepatic lipid metabolism dysregulation.

### 2.3. Olan Promotes Upregulation of ELOVL6 Gene Expression by Regulating the Transcription Factor HNF-3α

**Figure 5:**
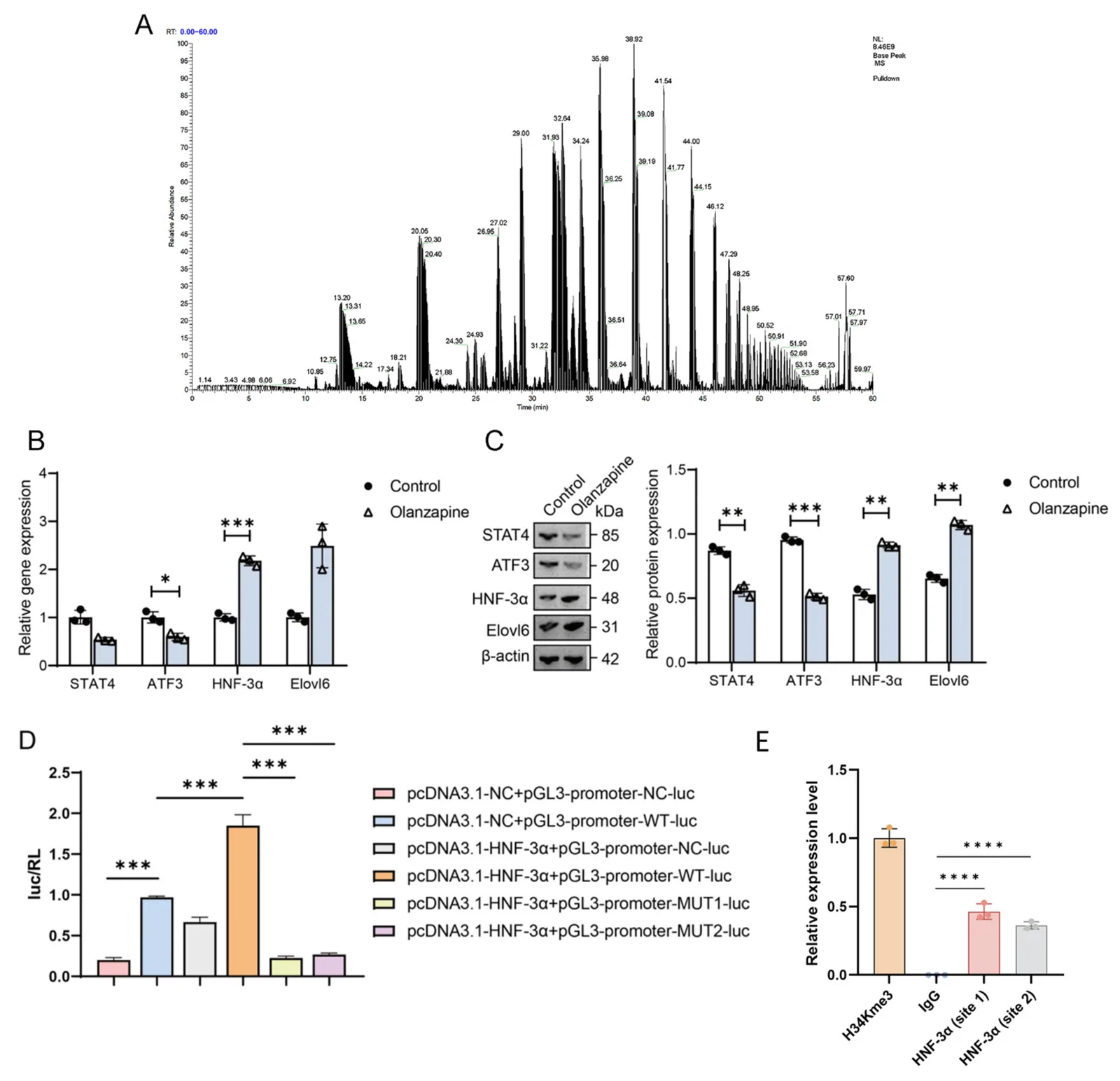
Transcription factor *HNF-3α* binds to the *ELOVL6* promoter. (**A**) Mass spectrometry analysis of proteins pulled down with *ELOVL6*. (**B**) qPCR analysis of the relative expression levels of pulled-down genes (*n* = 3). (**C**) Western blotting analysis of the relative expression levels of pulled-down proteins (*n* = 3). (**D**) Dual-luciferase assay validation of the direct binding of transcription factor *HNF-3α* to *ELOVL6* (*n* = 3). (**E**) ChIP-qPCR analysis of *HNF-3α* binding to the *ELOVL6* promoter in HL7702 cells (*n* = 3). * *p* < 0.05, ** *p* < 0.01, *** *p* < 0.001, **** *p* < 0.0001.

The molecular mechanism by which Olan regulates *ELOVL6* expression was investigated using DNA pulldown assays to screen for binding proteins within the *ELOVL6* promoter region. Mass spectrometry analysis identified three potential interactors: *STAT4*, *ATF3*, and *HNF-3α* (*FOXA1*) (Figure 5A,B). Western blot analysis revealed that Olan treatment significantly downregulated *STAT4* and *ATF3* expression, while upregulating *HNF-3α* and *ELOVL6* levels (Figure 5C). These findings suggest that *HNF-3α* may be closely associated with Olan-induced changes in *ELOVL6* expression.

To validate the direct regulatory role of *HNF-3α*, dual-luciferase reporter assays were performed. As shown in Figure 5D, *HNF-3α* overexpression markedly enhanced the luciferase activity of the wild-type *ELOVL6* reporter compared with the negative control (pcDNA3.1-*NC* + pGL3-promoter-*NC*-luc). In contrast, mutation of the predicted binding sites—either through deletion (MUT1) or point mutation (MUT2)—abolished *HNF-3α*-mediated activation. Collectively, these results demonstrate that *HNF-3α* directly binds to specific sites within the *ELOVL6* promoter to activate transcription.

This binding was further confirmed by ChIP-qPCR at two predicted promoter sites. As shown in Figure 5E, *HNF-3α* antibody exhibited significant enrichment at both sites relative to IgG controls, with H3K4me3 also showing robust enrichment. These data establish *HNF-3α* as a key transcription factor that directly binds the *ELOVL6* promoter and mediates Olan-regulated *ELOVL6* expression. Mechanistically, Olan promotes *ELOVL6* transcription by inducing *HNF-3α* expression and its subsequent recruitment to the *ELOVL6* promoter.

**Figure 6:**
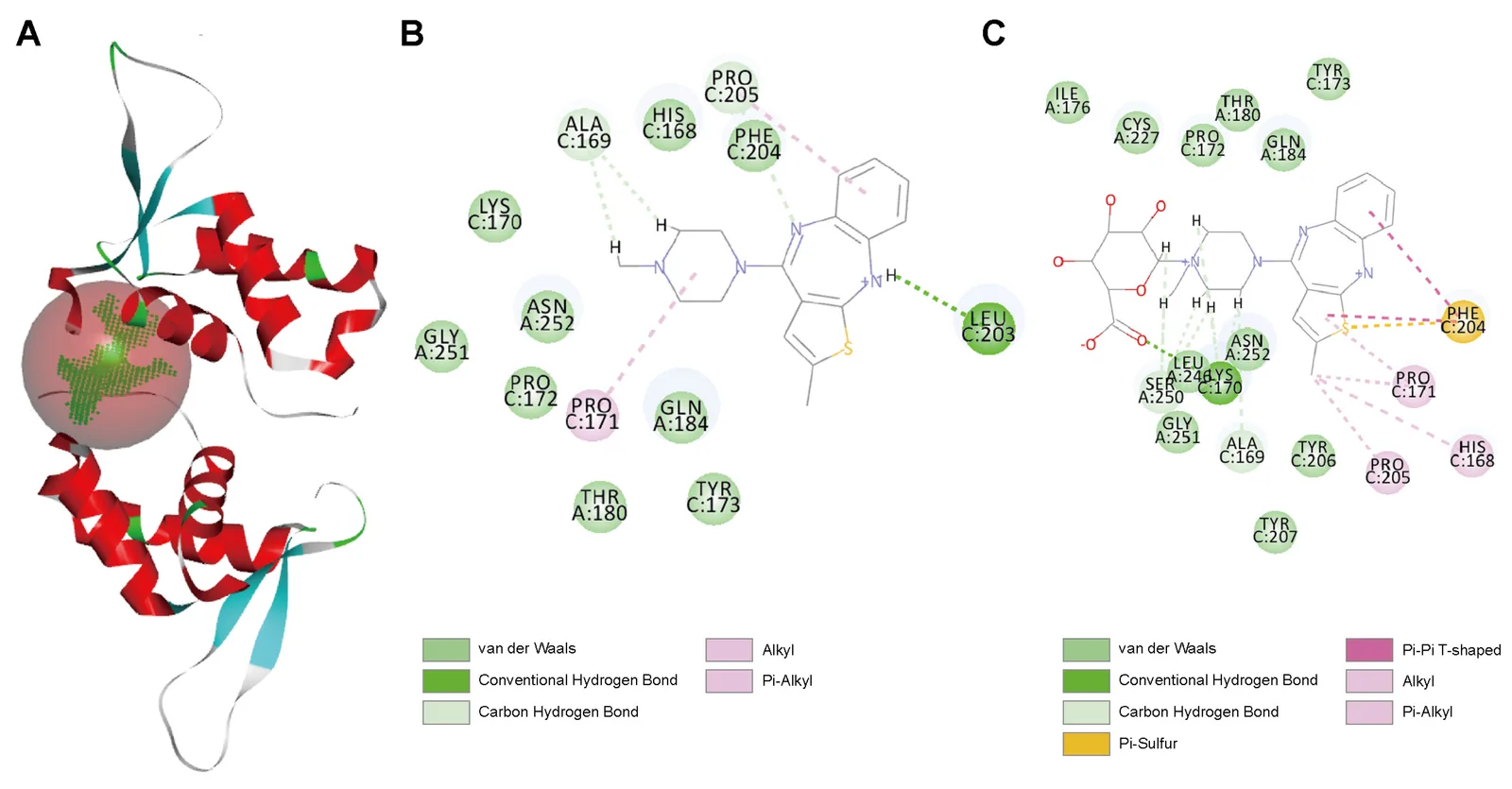
Molecular docking results of *HNF-3α* with Olan and its metabolite. (**A**) Three-dimensional structure of *HNF-3α* and visualization of potential binding pocket. Red helices: α-helices; Cyan arrows: β-sheets; White or light gray: coil/loop regions; Green: turn structures; Semi-transparent red spheres: binding site search region; Green dotted mesh: spatial sampling points of potential active sites. (**B**) Molecular interactions between Olan and *HNF-3α* protein. CDocker score: 3.06 kcal/mol. (**C**) Molecular interactions between Olan 4′-N-glucuronide and *HNF-3α* protein. CDocker score: −28.00 kcal/mol.

Finally, the interaction between Olan and *HNF-3α* was explored using molecular docking analysis, as shown in Table 1 and Figure 6A,B. Results indicated that Olan binds *HNF-3α* with a CDocker score of 3.06 kcal/mol, suggesting an energetically unfavorable interaction despite detectable hydrogen bonds, Pi–alkyl, and van der Waals contacts. Strikingly, however, all eight in vivo metabolites displayed energetically favorable binding. Notably, Olan 4′-N-glucuronide showed the most favorable binding energy (−28.00 kcal/mol), suggesting the highest affinity for *HNF-3α*. Detailed analysis revealed that Olan 4′-N-glucuronide forms hydrogen bonds or carbon–hydrogen bonds with residues LYS170, LEU246, and ASN252, Pi–Pi and Pi–sulfur interactions with PHE204, and Pi–alkyl and alkyl interactions with PRO171, HIS168, and PRO205 (Figure 6C). Taken together, these findings indicate that Olan likely regulates *ELOVL6* transcriptional activity indirectly through its metabolite Olan 4′-N-glucuronide binding to *HNF-3α*.

### 2.4. Association of Elovl6 Gene Single-Nucleotide Polymorphism (SNP) Variants with Weight Gain in Patients Taking Olan

The association between *ELOVL6* single-nucleotide polymorphisms (SNPs) and Olan-induced weight gain was investigated in 99 patients who experienced significant weight gain (>7% from baseline). High-throughput sequencing of the *ELOVL6* promoter revealed that 49 patients carried no mutations at the four examined SNP loci. Among the remaining 50 patients, the following mutations were identified: 15 patients exhibited C → T mutations at rs6824447 (statistically significant weight changes); 17 patients showed C → T mutations at rs3813830 (highly significant weight changes); 4 overweight patients harbored mutations at rs3813829; and 14 patients displayed T → C mutations at −1427 bp (Table 2). Collectively, these findings identify rs6824447, rs3813829, rs3813830, and −1427 bp as potential genetic risk loci for Olan-induced weight gain.

Gender differences in *ELOVL6* gene mutations were further explored through sex-stratified analysis of patients experiencing Olan-induced weight gain. Among the 99 patients with significant weight gain, the overall mutation rate at SNP loci in the *ELOVL6* gene promoter was 50.51%. Specifically, C → T mutations at the rs6824447 locus showed no significant differences between genders. Similarly, T → C mutations at the rs3813829 locus and mutations at the rs3813830 locus exhibited no significant gender distribution differences. In contrast, mutations at the −1427 bp locus occurred predominantly in female patients, demonstrating obvious gender specificity (Table 3). These results suggest that the −1427 bp locus mutation represents a potential genetic risk locus specifically for Olan-induced weight gain in female patients.

The exclusion of confounding factors was validated through multiple regression analysis, incorporating drinking history, family genetic history, endoplasmic reticulum stress markers, body mass index, gender, blood lipid levels, and waist-to-hip ratio as covariates. The results showed (Table 4) that among the four SNP loci in the *ELOVL6* gene promoter region, mutations at the rs6824447 locus were weakly associated with Olan-induced weight gain but showed no association with gender differences. Mutations at the rs3813829, rs3813830, and −1427 bp loci were all significantly associated with Olan-induced weight gain, with the −1427 bp locus mutation representing a potential genetic risk locus associated with gender differences in Olan-induced weight gain.

**Figure 7:**
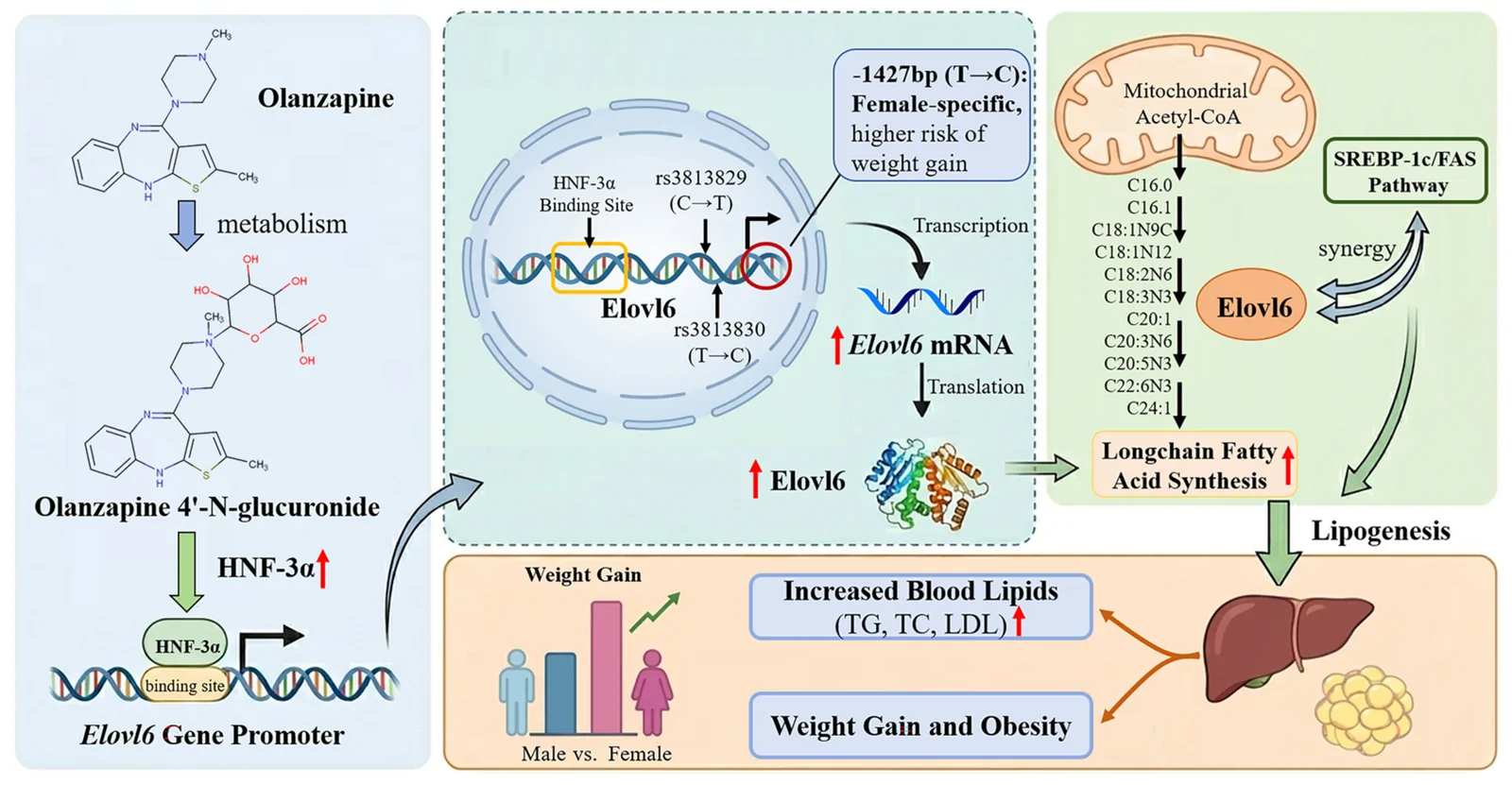
Hypothesis on the mechanism of weight gain induced by differential expression of *Elovl6* in olanzapine. TG: triglycerides, TC: total cholesterol, LDL: low-density lipoprotein.

In summary, our data support a model wherein Olan modulates *HNF-3α* signaling to upregulate *Elovl6*, thereby enhancing lipogenic gene networks and precipitating aberrant weight accumulation. Furthermore, SNPs in the *ELOVL6* promoter region, specifically rs3813829, rs3813830, and the -1427 bp variant, represent critical determinants of Olan-induced weight gain. Notably, the -1427 bp mutation exhibits female-specific characteristics and shows significant correlation with clinical weight gain. The proposed mechanism for Olan-induced weight gain is illustrated in Figure 7.

## 3. Discussion

### 3.1. Olan Upregulates Hepatic ELOVL6 to Promote Long-Chain Fatty Acid Synthesis in a Sex-Dependent Manner

Olan-induced obesity exhibits pronounced sex differences, with mechanisms involving sex-specific expression of key genes regulating hepatic fatty acid metabolism. To elucidate the underlying mechanisms, this study established a rat model through chronic Olan administration and conducted hepatic transcriptome sequencing to identify molecular determinants of sexual dimorphism in weight gain. Differentially expressed genes between female control and Olan-treated groups were intersected with those showing sex-specific responses to Olan, revealing *ELOVL6*, a gene encoding fatty acid elongase, as significantly upregulated in females. This finding aligns with Moon et al., who demonstrated that *ELOVL6* knockout reduces hepatic stearate (C18:0) and oleate (C18:1n-9) ratios in mice [23]. Functionally, *ELOVL6* catalyzes the conversion of palmitate (C16:0) to stearate and oleate to 20-carbon unsaturated fatty acids, serving as a key regulator of long-chain fatty acid composition [24,25,26]. Other studies have shown that *ELOVL6* can modulate the fatty acid profiles of goat mammary epithelial cells [27] and pig intramuscular adipocytes [28]. Metabolomic analysis from the present study confirmed elevated normalized intensities of long-chain fatty acids in Olan-treated females. Collectively, these findings indicate that Olan promotes lipogenesis through *ELOVL6* upregulation, thereby contributing to sex-biased adiposity and offering a potential molecular target for understanding gender disparities in antipsychotic-induced metabolic complications.

### 3.2. Olan Promotes Hepatic Lipogenesis Through ELOVL6-Mediated Transcriptional and Metabolic Mechanisms

Olan-induced metabolic disturbances involve intricate interconnections between obesity and insulin resistance, with hepatic fatty acid elongase *ELOVL6* emerging as a pivotal node linking these pathological states. Clinical observations indicate that insulin resistance frequently co-occurs with obesity, and hepatic fatty acid composition serves as a critical determinant of insulin sensitivity. Pharmacological studies have demonstrated that *ELOVL6* inhibition ameliorates insulin resistance independent of body weight reduction [29], whereas genetic ablation of *ELOVL6* attenuates metabolic disorders associated with insulin resistance in murine models [30]. To investigate whether Olan modulates hepatic lipogenesis through *ELOVL6*, this study established a cellular model of stable *ELOVL6* overexpression in *HL7702* hepatocytes. The results showed that Olan treatment markedly upregulated the levels of lipogenic genes and proteins, including *SREBP-1c* and *FAS*. This synergistic effect appears to derive from dual regulatory mechanisms. On one hand, Olan may activate *HNF-3α* (also known as *FOXA1*)-mediated transcriptional regulation, a factor that governs multiple metabolic genes associated with fatty acid synthesis [31]. On the other hand, *ELOVL6* upregulation directly facilitates the conversion of palmitate to stearate and the subsequent production of longer-chain fatty acids [32], whose accumulation may further activate *SREBP-1c* and *FAS*, establishing a positive feedback loop that amplifies de novo lipogenesis [33]. Additionally, altered fatty acid composition may indirectly modulate lipogenic gene expression through signaling pathways such as AMPK [34,35,36]. These findings corroborate prior studies on *ELOVL6*-mediated regulation of fatty acid profiles and collectively support a mechanistic model wherein Olan drives hepatic steatosis and insulin resistance through *ELOVL6*-dependent enhancement of lipid synthesis.

### 3.3. HNF-3α-Mediated Transcriptional Activation of ELOVL6 Underlies Olan-Induced Lipogenesis

Transcriptional regulatory mechanisms represent a critical entry point for dissecting *ELOVL6*-mediated lipid metabolic disturbances. Bioinformatic and transcriptomic analyses have demonstrated that *ELOVL6* overexpression systematically remodels the signaling networks of bovine adipocytes, with downregulated genes enriched in lipolysis regulation and Wnt signaling pathways, whereas upregulated genes predominantly involve FoxO, PI3K-Akt, and cAMP signaling cascades [37]. These observations suggest that *ELOVL6* governs lipid homeostasis through multi-pathway synergistic interactions. To elucidate the specific regulatory mechanisms of *ELOVL6* in Olan-induced lipogenesis, this study integrated DNP-pulldown, quantitative PCR, and Western blot analyses to screen for *ELOVL6*-interacting proteins. The results indicated that Olan treatment markedly upregulated transcription factor *HNF-3α* expression. Subsequent functional validation experiments revealed that *HNF-3α* directly bound to the *ELOVL6* promoter and enhanced its transcriptional activity, as evidenced by increased luciferase reporter signals. ChIP-qPCR further validated the direct interaction between *HNF-3α* and the *ELOVL6* promoter in HL7702 cells. Collectively, these findings indicate that Olan activates *HNF-3α*, thereby upregulating *ELOVL6* expression and establishing the *HNF-3α*/*ELOVL6* regulatory axis, ultimately contributing to sex-biased weight gain.

### 3.4. Genetic Variants in ELOVL6 Drive Sex-Specific Metabolic Responses

Genetic polymorphism analysis offers a translational clinical perspective for elucidating *ELOVL6*-mediated interindividual metabolic variability. Based on clinical specimens from 99 patients receiving Olan treatment, this study systematically screened four SNPs within the *ELOVL6* gene promoter region for associations with BMI changes, revealing that rs6824447, -1427 bp, rs3813830, and rs3813829 were all correlated with BMI fluctuations, with the -1427 bp locus exhibiting significant female-specific distribution. This genetic evidence converges with prior research: a prospective study in Southern Spanish populations previously reported an association between the rs6824447 locus and enhanced insulin sensitivity [38], whereas the present study, although observing an association between this locus and Olan-induced weight gain, demonstrated a modest effect size without sex-stratified characteristics, suggesting that this SNP may exert differential regulatory roles under basal metabolic homeostasis versus pharmacological stress conditions. Cross-species investigations further substantiate this inference; Corominas et al., through high-throughput sequencing analysis of the complete genetic architecture of porcine *ELOVL6*, identified the c.-394G>A variant as the most probable polymorphism on chromosome eight influencing differential *ELOVL6* expression, which modulates palmitic and palmitoleic acid content in muscle and backfat tissues [39]. Integrating these population-based association analyses with animal model evidence, genetic variations in the *ELOVL6* promoter region may regulate lipid metabolism in a sex-specific manner.

### 3.5. Limitations

There are some limitations. First, the molecular pathway identified in our research is based solely on animal and in vitro experiments, which may not entirely reflect actual human conditions. Second, the sample size used for the SNP analysis is relatively small. Third, molecular pathways of diseases are typically not singular; our transcriptomic sequencing and transcription factor predictions identified multiple target genes and transcription factors, yet our study focused solely on *Elovl6* and *HNF-α*. Fourth, while we identified changes in the transcriptional regulation of *Elovl6* by *HNF-α* and the presence of SNPs in the *Elovl6* promoter, we did not explore the relationship between these two findings. Future research should expand on investigating the molecular mechanisms of Olan-induced weight gain in females and increase sample sizes for validation in human subjects, which holds significant clinical implications for addressing Olan’s side effects.

## 4. Materials and Methods

### 4.1. Animals and Treatments

All experimental procedures involving animals were authorized by the Ethics Committee of Southwest University (Xixiao Yaolun Zi 2019-10). They follow the National Institutes of Health guidelines and strictly comply with ethical principles, laws, and regulations. Sprague–Dawley rats (8 weeks, 180–220 g) were obtained from Hunan SJA Laboratory Animal Co., Ltd. (Changsha, China) [SCXK (Xiang) 2019-0004].

**Figure 8:**
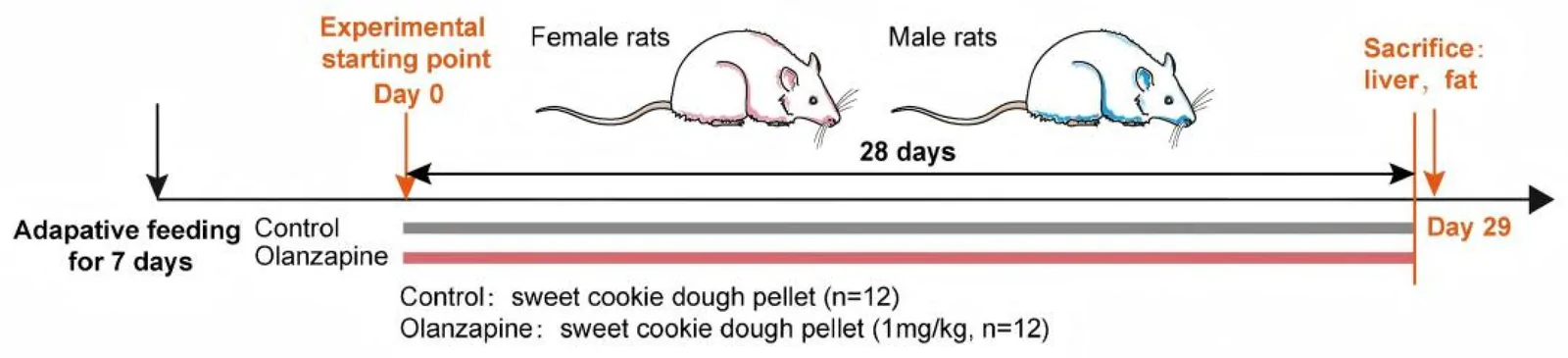
The plan of animal treatments.

As illustrated in Figure 8, rats were singly maintained under controlled conditions (22 ± 1 °C; 12 h light/dark cycle) with unrestricted access to chow and water. One week later, the rats were randomly divided into four groups using a random number table: the male control group, the male treatment group, the female control group, and the female treatment group. To ensure adequate statistical power and adhere to the “3R” principles, each group consisted of 6 rats, totaling 24 rats. Each cage housed two rats. During the experimental period, sweet cookie dough pellets (32% cornstarch, 30% sucrose, 30% water and 8% casein) were administered at 9:00 and 21:00 every day for 28 consecutive days (3 g/kg each day). The dough pellets for the treatment groups contained Olan (Olan dose: 1 mg/kg per administration). The amounts of chow and water provided to each rat were adjusted according to body weight and were kept consistent across groups. During feeding, an opaque divider was used to separate the two rats to prevent interference. After feeding, the divider was removed. The dough pellets were provided before regular food to ensure complete consumption of the pellets. If the food was not completely consumed within 20 min, the remaining feed was broken into smaller, palatable pieces and gently placed near the rat’s mouth to encourage consumption. If there was still leftover food 30 min after the beginning of feeding, the weight of the remaining food and pellets was recorded. The estrous cycles of female rats were monitored via vaginal smearing to exclude potential effects of cyclic metabolic fluctuations on the results. All experimental rats were housed under identical conditions, and rats that died during the experiment were excluded. On day 29, the cumulative consumption of pellets and food by each rat during the experiment was calculated, excluding rats with low intake (cumulative intake of either pellets or food of less than 90% of the target dose on ≥5 occasions). The average intake of the two rats per cage was considered the final result for that cage. Ultimately, all six rats in each group met the criteria and were included in the final analysis, with no significant differences in total intake of pellets or food among the groups. The rats were incubated in sodium pentobarbital (1% in normal saline, 2 mL/kg). After anesthesia, euthanasia was performed by cervical dislocation, and the entire process strictly followed the principles of animal welfare. Fat and liver tissue samples were preserved at −80 °C. Three samples were randomly selected from each group for subsequent transcriptomic and metabolomic analyses, while the remaining three served as independent samples for validation of differential gene and metabolite levels.

### 4.2. Transcriptome Sequencing of Fat and Liver Tissues

RNA was isolated from the fat and liver samples, while its concentration and purity were detected by agarose electrophoresis and an Agilent 2100 bioanalyzer (Agiloent technologies, Santa Clara, CA, USA). Eukaryotic transcripts were enriched using Oligo (dT) magnetic beads, followed by mRNA fragmentation using a fragmentation buffer, and then cDNA was synthesized using the fragmented mRNA as the template. After further purification by magnetic beads and elution with EB buffer, the eluted cDNA underwent end repair and A-tailing. Then, the fragment size was selected by magnetic beads, and U-containing chains were degraded for final library construction. Sequencing was carried out using an Illumina NovaSeq 6000 platform (PE150) (Illumina Inc., San Diego, CA, USA). Filtered sequencing data were obtained using the bowtie2 package (v2.5.3). The high-quality sequences were aligned to the reference genome to quantify gene-level expression. DESeq software (v1.40.2) in the R language was used to analyze mRNA differential expression with FDR < 0.05 and |log2FoldChange| > 1.0. Further, differentially expressed genes (DEGs) were clustered, the Pheatmap software (v1.0.13) package was used to conduct bidirectional cluster analysis, target genes were identified, and Kyoto Encyclopedia of Genes and Genomes (KEGG) and Gene Ontology (GO) analyses were performed.

### 4.3. Quantitative Real-Time Polymerase Chain Reaction (qPCR)

The collected liver tissues were ground for homogenization. RNA extraction was performed using the TRIzol method (9108, Takara, Kusatsu, Japan). Briefly, tissues were lysed in TRIzol reagent, followed by the addition of chloroform. After centrifugation, the supernatant was mixed with isopropanol to precipitate RNA. After another round of centrifugation, RNA pellets underwent ethanol washing and natural drying and were then solubilized in RNase-free water. cDNA synthesis was carried out with a reverse transcription kit (PrimeScript™II 1st Strand cDNA Synthesis Kit, 6210A, Takara, Kusatsu, Japan). qPCR was performed on an ABI 7500 Real-Time PCR System (Thermo Fisher, Waltham, MA, USA) with a SYB-Green QPCR kit (RR820A, Takara, Kusatsu, Japan). The reaction system consisted of 1.6 μL of primers (10 μM), 0.4 μL of ROX Reference Dye II, 2.6 μL of cDNA template, and 10 μL of TB Green Premix Ex Taq II (Tli RNaseH Plus). ddH_2_O was supplemented to reach a total volume of 20 μL. GAPDH was used as an internal reference. The reaction conditions were set as 95 °C for 5 s and 60 °C for 30–34 s for 40 cycles. The primer sequences are provided in Table 5. Gene expression levels were calculated by the 2^−ΔΔCt^ approach.

### 4.4. Western Blot

For cell samples, harvested cells were scraped by a cell scraper and then centrifuged at 12,000 r/min for 15 min. The resulting supernatant was retained. For tissue samples, tissues were first placed in a pre-chilled mortar and frozen with liquid nitrogen. The tissue was then thoroughly ground using a grinding rod. RIPA lysis buffer (Beyotime, Shanghai, China) was added to the samples for total protein extraction.

Sixty micrograms of denatured protein were separated by electrophoresis and transferred to a PVDF membrane (Millipore, Burlington, MA, USA). The membrane was then washed in TBST for 1 min and blocked with 5% non-fat milk for 1 h at room temperature. Subsequently, it was incubated at 4 °C overnight with primary antibodies against *ELOVL6* (Bioss, Beijing, China, bs-7055R, 1:1000), *STAT4* (Bioss, Beijing, China, bs-10023R, 1:1000), *ATF3* (Bioss, Beijing, China, bs-0519R, 1:1000), *HNF-3α* (Bioss, Beijing, China, bs-8634R, 1:1000), and *GAPDH* (Bioss, Beijing, China, bs-13282R, 1:1000) on a shaker. Following incubation with goat anti-rabbit IgG H&L (Bioss, Beijing, China, bs-0295G) for 1 h at room temperature, the membrane was washed and exposed to film for development. Images were captured using a gel imaging system (Bio-Rad, Hercules, CA, USA). Grayscale values and relative protein expression were quantified using ImageJ v1.54.

### 4.5. Liver Fatty Acid Metabolomics Analysis

Liver tissue samples of the control group and Olan group were collected, and a 2:1 chloroform–methanol mixture (1 mL) was added. Tissue homogenates were obtained using a high-throughput tissue grinder (MB-96, Mebi, Jiaxing, China). After centrifugation for 5 min (12,000 rpm, 4 °C), the supernatant was obtained. A 2 mL volume of 1% methanol sulfate solution was used for esterification at 80 °C for 30 min. After cooling, extraction was performed with 1 mL of n-hexane for 5 min, followed by washing with 5 mL of pre-cooled H_2_O, and centrifugation at 3500 rpm at 4 °C for 10 min. Then, 100 mg of anhydrous sodium sulfate powder was added to 700 μL of supernatant, shaken and mixed for 30 s, and centrifuged at 12,000 rpm for 5 min to remove excess water. After dilution of 100 μL of supernatant using 400 μL of n-hexane, 300 μL aliquots were supplemented with 15 μL of 500 ppm methyl salicylate serving as the internal reference. The composition and content of fatty acids in liver tissues were analyzed by liquid chromatography–mass spectrometry (LC-MS) targeted metabolomics with a gas chromatograph (Trace 1300, Thermo, MA, USA) and mass spectrometer (TSQ 9000, Thermo, MA, USA). The standard curve working solution was prepared by diluting a 51-fatty-acid methyl ester mixed solution (4000 µg/mL) with n-hexane to obtain 10 mixed standard concentration gradients of 1–2000 µg/mL. Chromatography was performed on a Thermo TG-FAME capillary column (0.20 µm, 50 m × 0.25 mm) with a 1 μL injection volume and a split ratio of 8:1. The carrier gas was helium with a 0.63 mL/min flow rate. Mass spectrometry was performed under electron impact (EI) ionization, using SIM mode with an electron energy set at 70 eV.

### 4.6. Construction of Lentiviral Vectors for ELOVL6 Gene Overexpression and Interference

The short hairpin (shRNA) interference targets were designed for the 5 ‘-GCTCTGTATGCTGCTTTAT-3′ transcription sequence of the *Elovl6* gene. The oligo fragments purified by PAGE gel were synthesized and diluted to 100 μM, respectively. They were combined into Oligo+(5′-CACCGCTCTGTATGCTGCCTTTATACGAATATAAAGGCAGCATACAGAGC-3′) and Oligo-(5‘-AAAAGCTCTGTATGCTGCCTTTATATTCGTATAAAGGCAGCATACAGAGC-3′) primers, which were each diluted to 100 μM. After the annealing reaction, double-stranded pLVX-shRNA1 expression vectors were formed and recovered by enzymatic digestion to obtain pure linearized vectors. Overnight ligation at 4 °C facilitated the transformation of the pLVX-shRNA 1-sh*Elovl6* vectors into DH5α competent cells, and the successful construction was verified by sequencing.

The PCR-amplified *ELOVL6* was cloned into the Xba I/BamH I restriction site of the pCDH-CMV-MCS-EF1-copGFP-T2A-Puro vector, generating the *ELOVL6* expression vector. After restriction digestion verification and sequencing confirmation, it was transformed into the Escherichia coli DH5 α strain for amplification and purification. Finally, the plasmid containing the correct insertion fragment was selected through restriction digestion and sequencing confirmation.

The packaged lentivirus used for *Elovl6* interference/overexpression was introduced into HL 7702 cells. After 48 h of culture, a medium containing 2 mg/L purinomycin was added. The medium was changed every 2 days until the cells in the control group were completely dead. The screened cells were used for subsequent experiments.

### 4.7. HL7702 Cell Culture

HL7702 cells (#JY034) were provided by Shanghai Jinyuan Biotechnology Co., Ltd. (Shanghai, China). The cells were cultured in MEK medium (basal medium, L110KJ, Shanghai, China). Before use, the medium was supplemented with 10% fetal bovine serum (Life-iLad, AC031L055, Shanghai, China) together with 1% penicillin–streptomycin (Beyotime, C0009, Shanghai, China) to make a complete culture medium. Cells were routinely stored at 37 °C and 5% CO_2_, with medium renewal every 2 days. For Olan treatment and concentration determination, upon reaching 80–90% confluence, cells underwent trypsin digestion (Beyotime, Shanghai, S310KJ, China) and then were subjected to passaging. Referring to previous studies [40,41,42], HL7702 cells at the logarithmic phase were treated with Olan at concentrations of 1, 10, and 20 μmol/L, with a solvent control group set up. The expression level of the *ELOVL6* gene was detected after 24 h to determine the optimal drug concentration for subsequent experiments.

### 4.8. CCK-8

Cells were seeded into 96-well culture plates at a density of 5000 per well. Blank wells received an equal volume of cell culture medium. Each well received 10 μL of CCK-8 reagent (C0038, Beyotime, Shanghai, China) followed by 1 h incubation. A microplate reader was used to detect the absorbance values at 450 nm, and cell viability (%) was calculated as: (ODsample − ODblank)/(ODcontrol − ODblank) × 100%.

To determine the IC50 of Olan in HL7702 cells, after cell adhesion, cells were treated according to the following groups: the control group was cultured normally in DMEM/F12 complete medium, and the experimental groups were maintained in media supplemented with various concentrations of Olan (5, 10, 20, 40, 80, and 160 μM). Samples were collected for the CCK-8 assay after 24 h. Data were imported into GraphPad Prism software 8.0, and IC50 was calculated using “log(inhibitor) vs. normalized response—Variable slope” curve fitting.

### 4.9. Cell Grouping

For experimental purposes, stable cell lines with *ELOVL6* overexpression or interference were collected and expanded. The following experimental groups were established: HL7702 cells transfected with sh-Con + OE-con (control group for *ELOVL6* interference and overexpression), sh-Con + OE-*ELOVL6*, sh-*ELOVL6* + OE-Con, sh-Con + OE-*ELOVL6* + Olan, sh-*ELOVL6* + OE-Con + Olan, and sh-Con + OE-Con + Olan. During cell culture, each group received 20 μmol/L Olan, 200 μL of 20% medical-grade fat emulsion + 800 μL of DMEM medium (Basal Media, L110KJ, Shanghai, China) containing 10% FBS (Life-iLad, AC031L055, Shanghai, China) (1 mL total). After 48 h of culture, the cells were collected for subsequent experiments.

### 4.10. Oil Red O Staining

HL7702 cells were plated in 12-well plates and maintained in the logarithmic growth stage. The culture medium was then aspirated, and each well received 100 µL of 20% medical fat emulsion modeling solution plus 400 µL of DMEM containing 10% FBS. After 24 h incubation, Oil Red O staining was performed to observe the differences in lipid formation among groups. For staining, the induced modeling cells were rinsed once with PBS and fixed with 4% paraformaldehyde for 30 min at room temperature. After discarding the fixative, the cells were washed three times with PBS. An Oil Red O working solution was applied, followed by a 30 min incubation. Following two washes with PBS, the cells were visualized and imaged using an inverted biological microscope (BLD-200, Cossim, Beijing, China).

### 4.11. DNA Pulldown Assay

First, using whole-genome DNA as a template, a specific sequence in the *ELOVL6* promoter, which contains potential transcription factor binding sites, was amplified by PCR. PCR was performed using biotin-labeled primers: the forward primer was *ELOVL6*-F: 5′-CAACAGAGGGAGACATCATCT-3′, and the reverse primer was *ELOVL6*-R: 5′-GAAAGGGCTGGGTTAGGTGGA-3′. The amplified products were confirmed by gel electrophoresis and then purified with a gel extraction kit to prepare biotin-labeled DNA probes. Subsequently, according to the kit instructions, streptavidin magnetic beads were incubated with the probes to construct probe–magnetic bead complexes. Nuclear proteins were extracted from the human hepatocyte cell line HL7702 and incubated with the complexes at 4 °C for 1 h to allow proteins to bind to specific DNA sequences. After washing and elution, protein samples were collected. Peptides in the pulled-down products were identified by mass spectrometry, resulting in the screening of three candidate transcription factors. Their expression levels were further analyzed by qPCR and Western blotting.

### 4.12. Dual-Luciferase Reporter Assay

The StarBase database (https://starbase.sysu.edu.cn/index.php) (accessed on 2 July 2022) was used to predict the target sequence linking *ELOVL6* with hepatocyte nuclear factor-3α (*HNF-3α*). The original sequence of the *ELOVL6* promoter containing the *HNF-3α* binding site was designated as the wild-type (WT) sequence, while the *ELOVL6* promoter sequence with a mutated *HNF-3α* binding site was designated as the mutant (MUT) sequence. The WT sequence or MUT sequences (MUT1: the predicted binding site sequence on the *ELOVL6* promoter was deleted; MUT2: partial base mutations were introduced into the predicted binding region) were inserted into the pGL3-promoter vector to construct luciferase reporter vectors. 293T cells were seeded into 96-well plates. Using 0.3 μL of transfection reagent (Biomedicine, Chongqing, China), the pGL3-promoter-MUT1-luc, pGL3-promoter-MUT2-luc, or pGL3-promoter-WT-luc vector was co-transfected into the cells together with either pcDNA3.1-*NC* (empty vector control) or pcDNA3.1-*HNF-3α* (*HNF-3α* overexpression vector), as well as the pRL-TK reference vector (expressing Renilla luciferase). At 48 h post-transfection, cells were collected and lysed. Firefly and Renilla luciferase activities were sequentially measured using the Dual-Luciferase Reporter Assay System (Promega, Madison, WI, USA). The ratio of these two activities was calculated as the relative activity of the reporter gene to eliminate differences in transfection efficiency.

### 4.13. ChIP-qPCR

When HL7702 cells reached the logarithmic growth phase, experiments were performed following the protocol of the Millipore Magna ChIP™ Kit: Cells were cross-linked with 1% formaldehyde for 12 min at room temperature, which was terminated with 0.125 M glycine. Cells were sequentially treated with the kit-supplied Cell Lysis Buffer and Nuclear Lysis Buffer, and chromatin was sonicated into fragments of 200–500 bp. A 10% aliquot of fragmented chromatin was reserved as the input group, while the remaining sample was divided and incubated with ChIP-grade H3K4me3 antibody (positive control), normal rabbit IgG (negative control), or *HNF-3α* antibody (experimental group) with gentle rotation at 4 °C overnight. The next day, Protein A/G magnetic beads were added and incubated for 1.5 h at room temperature to capture the “antibody–transcription factor–DNA” complexes. Three sequential washes of the magnetic beads were performed using the kit’s low-salt wash buffer, high-salt wash buffer, and LiCl wash buffer. Subsequently, elution buffer was added, and the system was incubated for 4 h at 65 °C to achieve protein–DNA decrosslinking. After that, Proteinase K was added, and incubation was carried out for 1 h at 37 °C to digest residual proteins. Purified DNA was recovered using the kit’s DNA purification columns. For qPCR, the SYBR Green Mix was used to detect the *ELOVL6* promoter binding region (Site 1: F 5′-GACCTGGAGTGGCTGAAGAA-3′, R 5′-CAGGGAGGTGGTGAAGAGTT-3′; Site 2: F 5′-CTGGTTCCTGCTGCTACCTT-3′, R 5′-GCTGCTGAAGTCCTGGTCTT-3′), and all grouped samples were amplified in the same batch. Relative expression levels were calculated with the positive control group as the reference.

### 4.14. Molecular Docking

To detect whether there is an interaction between Olan and the *ELOVL6* transcription factor *HNF-3α*, the interaction relationship between Olan and *HNF-3α* was predicted through molecular docking technology.

(1) Structural Preparation: The crystal structure of *HNF-3α* was downloaded from the PDB database (https://www.rcsb.org/) (PDB ID: 7VOX) (accessed on 12 December 2022). Following removal of water molecules, addition of hydrogen atoms and Momany–Rone charges, and setting of the protonation state at pH = 7.4, the structure of *HNF-3α* was energy-minimized using the CHARMm force field before molecular docking. The 3-D structures of Olan and its eight metabolites, i.e., 2-hydroxymethyl Olan, Olan 4′-N-glucuronide, Olan N-oxide, N-desmethyl-2-hydroxymethyl Olan, N-desmethyl Olan, 2-carboxy Olan, N-desmethyl-2-carboxy Olan, and Olan 10-N-glucuronide, were obtained from the PubChem database. The ionization state of the ligands was specified using a pH-based method. Before molecular docking, all ligands were energy-minimized using the CHARMm force field and Momany–Rone charges. The RMS gradient was set to 0.01 kcal/mol/Å2.

(2) Molecular Docking: CDOCKER was performed using BIOVIA Discovery Studio 2019 software. The potential pocket of *HNF-3α* was identified using the Define and Edit Binding Site module. Herein, the largest pocket with a volume of 100.375 Å3 was selected for the subsequent docking research. The number of starting random conformations of ligands was set to 20, and other parameters were set to default values.

### 4.15. Clinical Sample Collection for Olan-Induced Weight Gain

The participants were enrolled in a clinical observational study focused on weight gain induced by Olan treatment. Specifically, patients diagnosed with first-episode or recurrent schizophrenia spectrum disorders by two professional psychiatrists, according to the Diagnostic and Statistical Manual of Mental Disorders-V, were included. These patients received Olan treatment (at flexible doses) based on their clinical condition. The inclusion criteria specified an age range of 30–35 years. Exclusion criteria were as follows: (1) presence of somatic diseases (such as hyperlipidemia, diabetes, hypertension, cerebral infarction, and heart disease); (2) narrow-angle glaucoma, epilepsy, or febrile seizure; (3) previous use of long-acting antipsychotic formulations; (4) regular use of clozapine one month prior to enrollment or any antipsychotic medication within one month for recurrent patients; (5) electroconvulsive therapy (ECT) within one month prior to enrollment, presence of severe suicidal ideation or agitation; (6) abnormal laboratory findings; (7) history of drug-induced malignant syndrome, including parkinsonism and tardive dyskinesia; (8) pregnant or planning to become pregnant and lactating women; (9) poor response to three or more antipsychotic drugs with different mechanisms; and (10) contraindications to Olan use. A total of 99 eligible participants were enrolled in the study (43 males and 56 females). Fasting venous blood samples were collected 24 weeks post-treatment, centrifuged to obtain plasma, and stored at −80 °C. The study was approved by the Ethics Committee of the Affiliated Hospital of North Sichuan Medical College (No. 2023050ER-1). Written informed consent was obtained from all participants.

### 4.16. Extraction and Sequencing of Whole Blood DNA

Genomic DNA was extracted from plasma using the DNA Blood Mini Kit (AG20231, Accurate Biology, Changsha, China). Four haplotype-tagging single-nucleotide polymorphisms (tagSNPs) were selected from the HapMap database (http://www.1000genomes.org/): rs3813829, rs3813830, rs6824447, and −1427 bp. These SNPs have a minor allele frequency greater than 5% and an average spacing of approximately 330 kb, spanning 1427 kb of the *Elovl6* promoter, which constitutes about 50% of its length. PCR amplification was performed using the following primers:

*Elovl6*-F: 5′-TGATTATTCACAGGCTCAGGC-3′
*Elovl6*-R: 5′-CAGAAGTAATCCCAGCACTTT-3′

The PCR products were subjected to agarose gel electrophoresis, and the target DNA bands were excised. The target DNA was then recovered using a gel extraction kit (TianGen, Beijing, China) and subsequently subjected to Sanger sequencing.

### 4.17. Statistical Analysis

Data obtained from the experiments were analyzed using Graphpad 8.0. All experiments were conducted in triplicate, and the results from normally distributed data were presented as mean ± standard deviation. An independent-samples *t*-test was utilized for between-group analyses, whereas one-way Analysis of Variance (ANOVA) was employed for comparisons among multiple groups, followed by Tukey’s HSD post hoc multiple comparison test. A *p*-value < 0.05 was considered statistically significant.

## 5. Conclusions

This work systematically investigated the effects of chronic Olan administration on metabolic regulation in rats, emphasizing the sex-specific mechanisms underlying Olan-induced weight gain. Results showed that female rats exhibited significant body weight elevation following Olan treatment. Transcriptomic sequencing revealed marked upregulation of *ELOVL6* expression in hepatic tissue, specifically in females. Functional experiments demonstrated that *ELOVL6* overexpression promotes fatty acid synthesis through activation of the *SREBP-1c*/*FAS* axis, ultimately leading to lipid accumulation. Subsequent mechanistic investigations indicated that *HNF-3α* directly binds to the *ELOVL6* promoter to enhance its transcription, establishing a novel “Olan–*HNF-3α*–*ELOVL6*–lipogenesis” regulatory axis. Furthermore, from a clinical translational perspective, a T → C mutation at the −1427 bp position within the *ELOVL6* gene promoter was significantly associated with weight gain in female patients, suggesting that this locus represents a potential genetic biomarker for individualized therapeutic strategies. Collectively, this study provides evidence delineating the sex-specific regulatory mechanism whereby the “Olan–*HNF-3α*–*ELOVL6*–fatty acid synthesis” axis mediates Olan-induced weight gain, establishing *ELOVL6* as a critical effector molecule in this process. Targeted interventions against *ELOVL6* may offer a novel therapeutic strategy and serve as a precision medicine target for managing Olan-associated weight gain and improving patient treatment adherence.

## Figures and Tables

**Table 1:** The docking scores of Olan and eight metabolites.

Compounds	CDocker Scores (kcal/mol)
2-carboxy Olan	−7.14
2-hydroxymethyl Olan	−2.67
N-desmethyl Olan	−1.82
N-desmethyl-2-carboxy Olan	−7.47
N-desmethyl-2-hydroxymethyl Olan	−0.92
Olan 4′-N-glucuronide	−28.00
Olan 10-N-glucuronide	−12.62
Olan N-oxide	−1.19
Olan	3.06

**Table 2:** Effects of somatotype on SNP locus gene mutations.

SNP Site	Mutant Genotype		Somatotype		*p*-Value
		Medium	Obese	Overweight	
No mutation	-	31	12	6	
rs6824447	C → T	4	4	7	0.0462
rs3813830	C → T	0	6	11	<0.0001
−1427 bp	T → C	0	4	10	<0.0001
rs3813829	T → C	0	0	4	0.0087

SNP: single-nucleotide polymorphism.

**Table 3:** Impact of sex on SNP variant frequency.

SNP Site	Mutant Genotype	Sex		Mutation Frequency	*p*-Value
		Male (n, %)	Female (n, %)		
No mutation	-	26 (59.09)	23 (41.82)	49.49%	
rs6824447	C → T	8 (18.18)	7 (12.73)	15.15%	0.5812
rs3813830	C → T	5 (11.36)	12 (21.82)	17.17%	0.1843
−1427 bp	T → C	1 (2.27)	13 (23.64)	14.14%	0.0028
rs3813829	T → C	3 (6.82)	1 (1.82)	4.04%	0.194

**Table 4:** Association analysis of SNP variants with body mass index and sex.

SNP Site	Mutant Genotype	Pearson (Somatotype)		Pearson (Sex)	
		Correlation Coefficient	*p*-Value	Correlation Coefficient	*p*-Value
rs6824447	C → T	0.2147	0.0348	−0.0678	0.5092
rs3813830	C → T	0.4823	<0.0001	0.1364	0.1804
−1427 bp	T → C	0.5216	<0.0001	0.3015	0.0026
rs3813829	T → C	0.232	0.021	−0.131	0.196

**Table 5:** Primer sequences for detected genes.

Gene Name	Sequence 5′-3′
*Rat-ELOVL6*	Forward: CTGGTCACTGACTCTTGCGG Reverse: TAGTTCGGGTGCTTTGCTGA
*Rat-Slc37a2*	Forward: GCTGCGTCATGCTGATCTTG Reverse: TTAGGGCCAGACTAGAGCCA
*Rat-Slc37a2*	Forward: TTGGCTGCTCCCATGATGTT Reverse: AAATACTATCGCGGGTGGGT
*Rat-Acacb*	Forward: CGAGTTGGGAAGAAGTGCCA Reverse: AGGCCCAAGGTGTCATAAGC
*Rat-Aacs*	Forward: TGTCTCCGCAGCTAAAGCC Reverse: CCTTGGGAGGACATAGGGGA
*Rat-Vdr*	Forward: CCATTCAGGACCGCCTATCC Reverse: AGGAGAGGGAGCGGTATTGT
*Rat-LPL*	Forward: TCCTACTTCCGCTGGTCAGA Reverse: CCGGCTTTCACTCGGATCTT
*Rat-Oas1g*	Forward: TCTGGTTCCAGCTTTAGGCA Reverse: TGACTTGCCCTTGAGTGTGG
*h-ELOVL6*	Forward: AAGCACCCGAACTAGGAGATAC Reverse: CCCGCAAGGCATAGTAAGAG
*h-STAT4*	Forward: CTATGATGACAACTTTCCCATGG Reverse: GAAGAAGAATCGTTGCCATGG
*h-ATF3*	Forward: TGGGTCACTGGTGTTTGAGG Reverse: GATTCCAGCGCAGAGGACAT
*h-HNF-3alpha*	Forward: GAACACCATGACTACGAGCG Reverse: CTACTGCGCCGGGACTCAGG
*Rat-GAPDH*	Forward: CTGGGCTACACTGAGCACC Reverse: AAGTGGTCGTTGAGGGCAATG

## Data Availability

The original contributions presented in this study are included in the article. Further inquiries can be directed to the corresponding authors.

## Supplementary Materials

The following supporting information can be downloaded at https://www.mdpi.com/article/10.3390/ph19081223/s1, Figure S1: HE staining of liver tissues from male and female rats after Olan administration or not.
