# Supplementary material for: ELOVL6: A Key Factor in the Gender Differences of Olanzapine-Induced Weight Gain
Source: Pharmaceuticals (Basel). 2026 Aug 4;19(8):1223. doi: 10.3390/ph19081223 (PMC13516770; doi:10.3390/ph19081223)
Supplement: Supplementary file 1 [file pharmaceuticals-19-01223-s001.zip › pharmaceuticals-4402602-supplementary.pdf]

The following are the results of HE staining of liver tissues from male and female rats after Olan administration or not. Green arrows: inflammatory cells; Red arrows: congestion; Yellow arrows: lipid droplets; Blue arrows: necrosis.

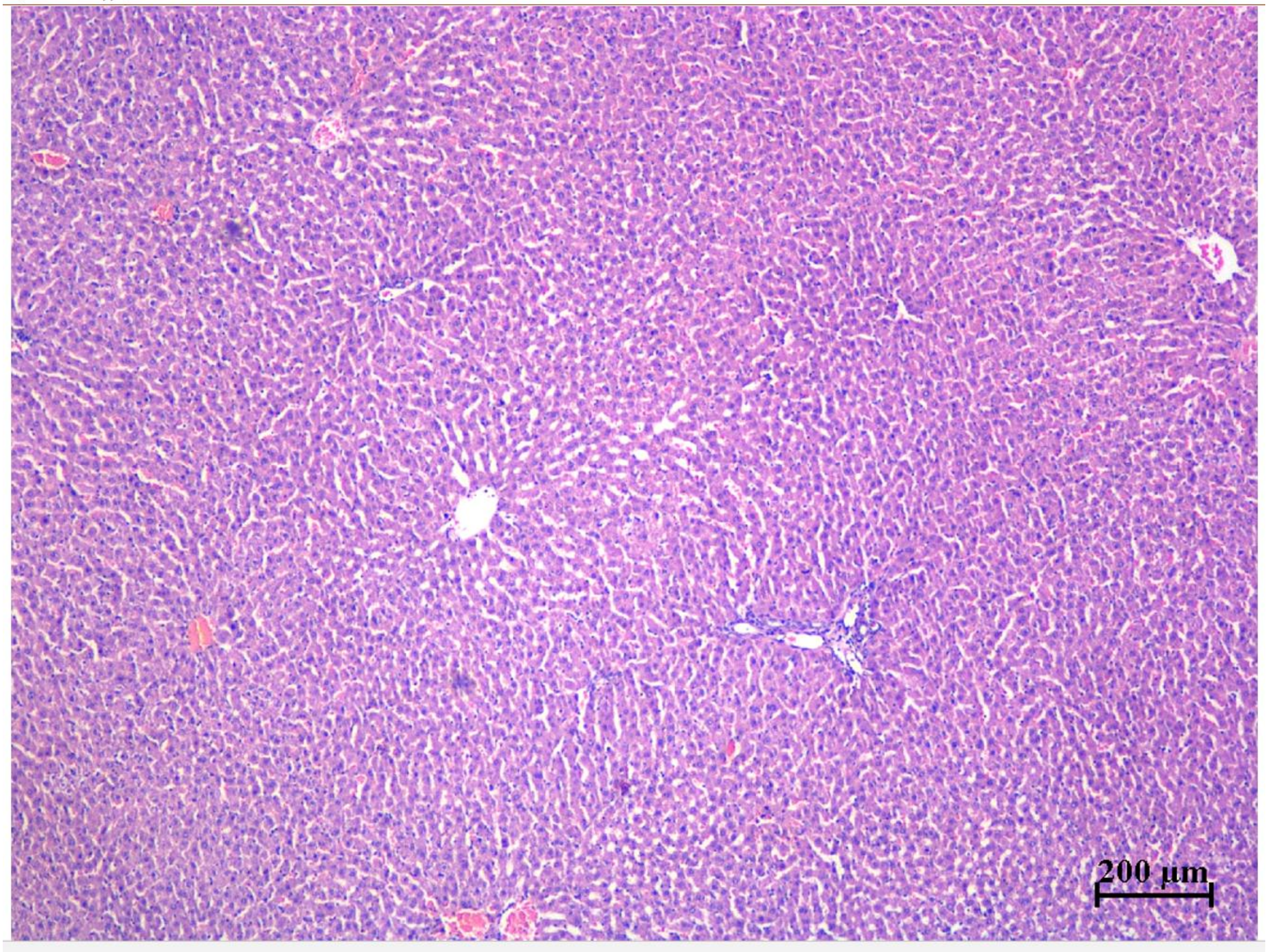

Male Control

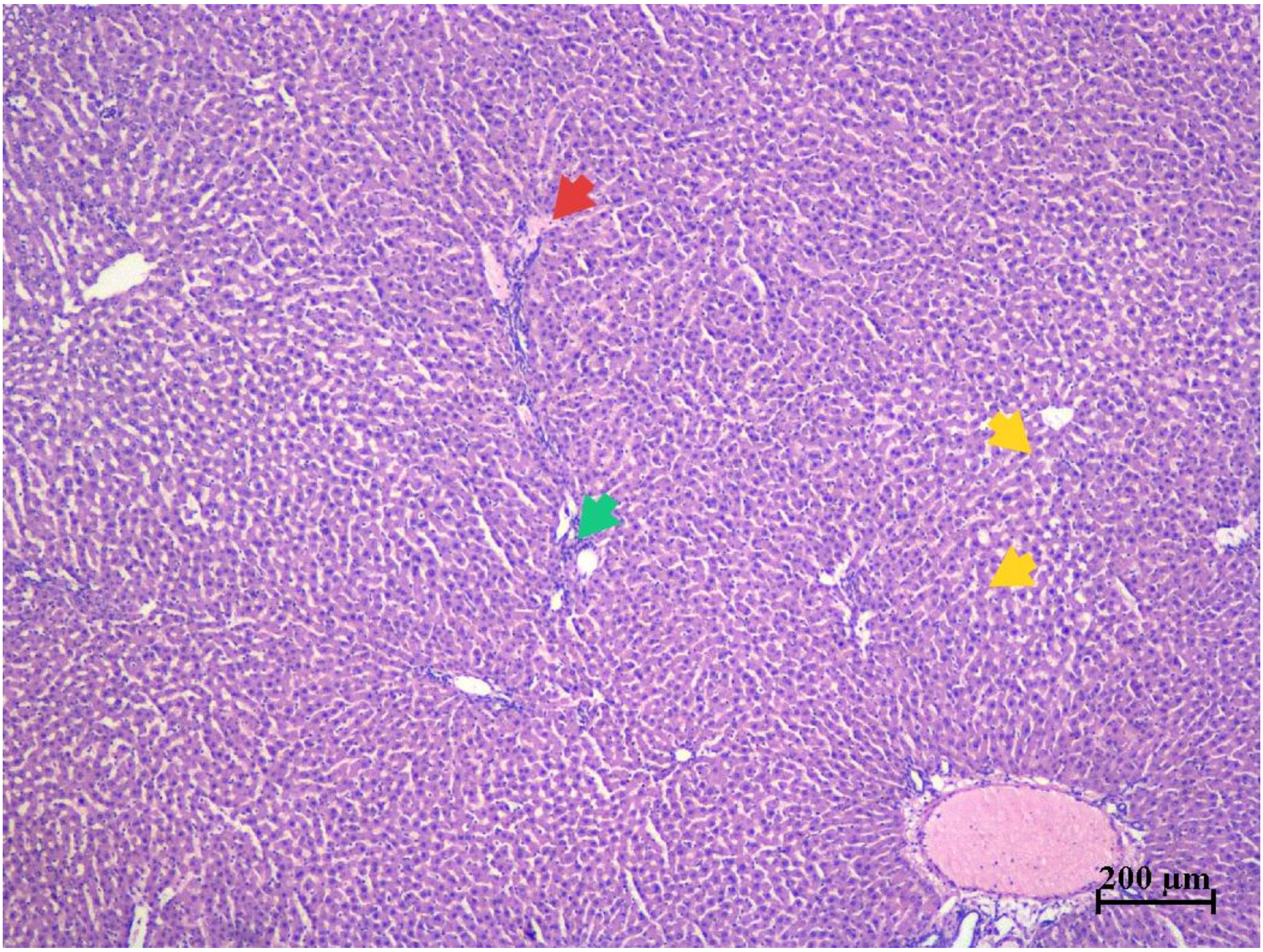

Male Olan

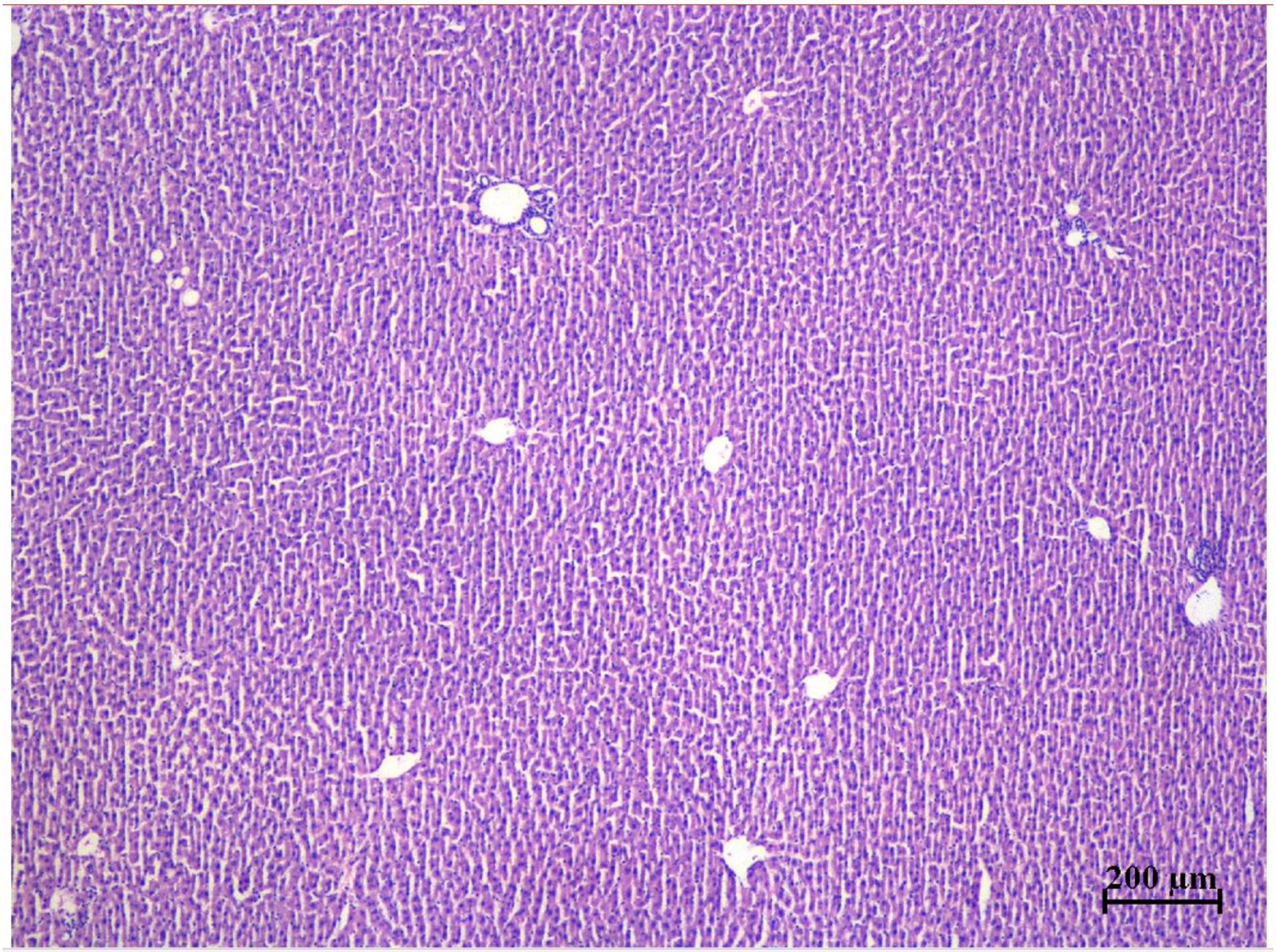

Female Control

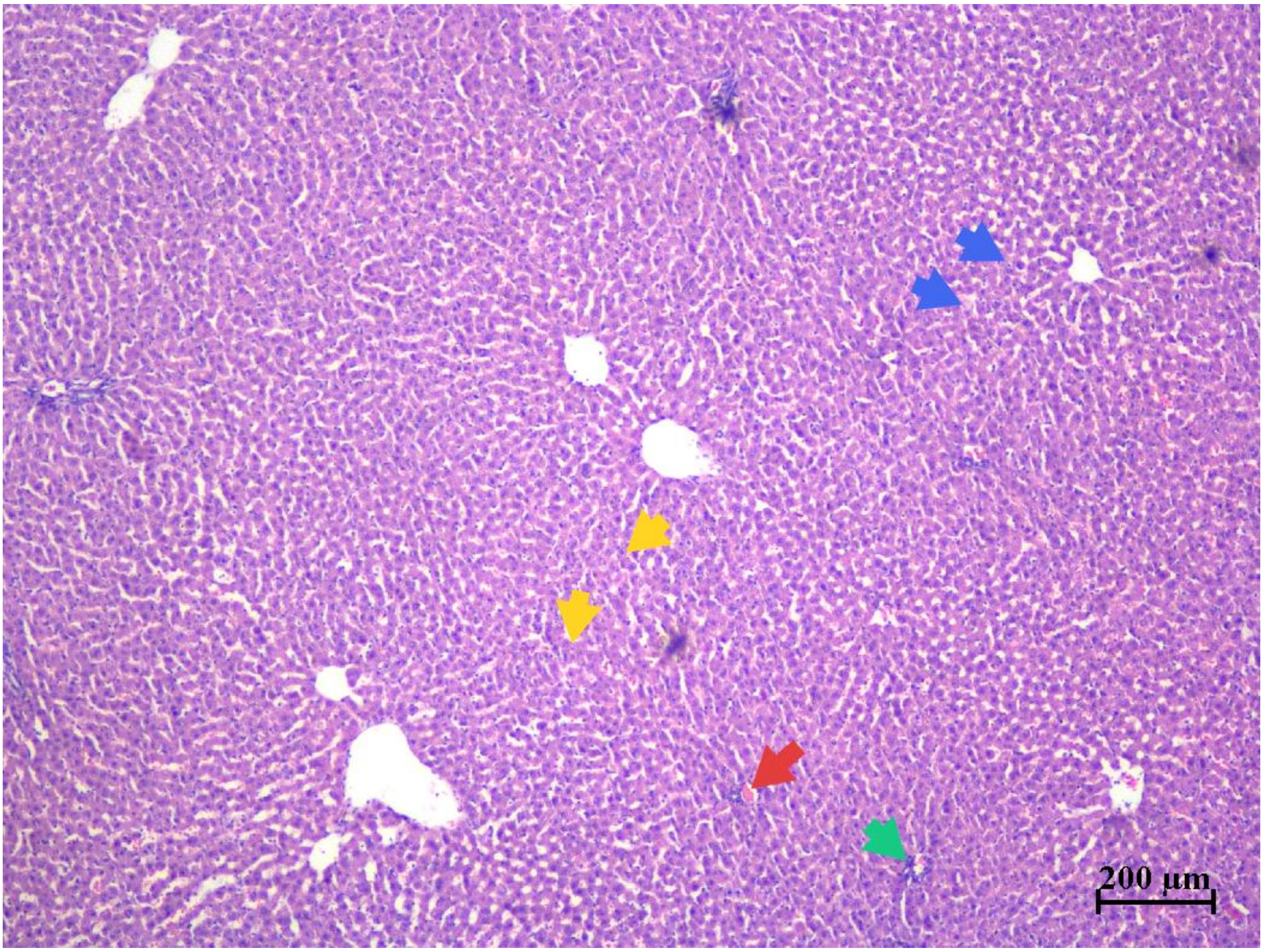

Female Olan

Figure S1. HE staining of liver tissues from male and female rats after Olan administration or not.
